# Chitosan impregnated sugarcane bagasse biochar for removal of anionic dyes from wastewater

**DOI:** 10.1038/s41598-024-77708-9

**Published:** 2024-11-07

**Authors:** Magda A. Akl, Asmaa A. Serage

**Affiliations:** Department of Chemistry, Faculty of Science, Mansoura University, Mansoura, 35516 Egypt

**Keywords:** Sugarcane bagasse, Biochar, Chitosan, Cong Red, Adsorption, Chemistry, Materials science

## Abstract

Wastewater treatment is of utmost importance in providing all equitable and safe drinking water. In the present study, a chitosan impregnated sugarcane bagasse biochar SCNC biocomposite has been synthesized for the removal of Congo red (CR) dye from an aqueous solution. The SCNC biocomposite was thoroughly characterized through Brunauer, Emmett and Teller (BET) and N_2_ adsorption isotherm, point of zero charge (pH_PZC_), elemental analysis, Scanning electron microscopy (SEM), Fourier transform infrared spectroscopy (FTIR) and Thermogravimetric analysis (TGA) analysis. Moreover, SCNC biocomposite was further employed to remove CR dye from the aqueous solution in batch mode. The SCNC biocomposite could remove more than 95.0% of CR at an initial concentration of (100mgL^− 1^), adsorbent dosage (0.05 g), time (200 min), pH ~ 3. The SCNC biocomposite achieved maximum adsorption capacity of 170mgg^− 1^. The equilibrium adsorption data for CR dye were best fitted to the Langmuir isotherm model with R^2^, 0.999. The kinetic and isotherm were statistically investigated using the chi-square statistic (χ2 ), mean square error (MSE), and the sum of squares error (SSE) Because of the higher correlation coefficient (R^2^ ≥ 0.999) and lower error functions, the equilibrium CR adsorption isotherms for a single-dye system fit Langmuir and the PSO kinetic model. The thermodynamic studies revealed the spontaneous and endothermic nature of adsorption of CR dye onto SCNC biocomposite. The SCNC biocomposite can be regenerated up to the 5th cycle successfully. The mechanism of CR adsorption onto SCNC was elucidated.

**Supplementary Information:**

The online version contains supplementary material available at 10.1038/s41598-024-77708-9.

## Introduction

Water pollution has become the most important topic that faces the whole of the world because of decreasing in different water resources. One of the most dangerous sources of water pollution is synthetic dyes. Textile industries such as paper, leather and plastics discharge large amounts of colored wastewater containing various dyes, which are mutagenic and carcinogenic to human beings^1,2^. Synthetic dyes are very stable against light, heat and oxidizing agents that made them have highly fatal effect to aquatic systems especially that they are non-biodegradable^3,4^. The danger of some dyes lies in causing severe diseases such as, cancer, skin irritation, allergic dermatitis,, and may result in human mutation^5,6^. Such dyes toxicity comes from the degradation to form toxic aromatic amines which can cause cancer in human beings^7^.

Congo Red (CR) is an anionic dye and is one of the synthetic dyes present in wastewater. It is sodium salt of benzidinediazobis-1-naphthylamine-4-sulfonic acid azo dye with high solubility in natural aqueous solutions^6^. It is very important to remove such type of dyestuffs from water resources before discharging into natural water because such effluents cause coloration of water and prevent sunlight from reaching to photosynthetic bacteria and aquatic environment causing big damage^8^.

Several analytical processes have been used for wastewater treatment such as adsorption, coagulation–flocculation, oxidation–ozonation, reverse osmosis, membrane filtration, biological degradation and electrochemical processes and nanocomposites^9–18^. Recently, several nano-composites have been prepared and used for wastewater treatment^19–22^. The adsorption technique has been chosen for the removal of dyes because of high efficiency, simplicity and easy reusability of the adsorbent^9,23^.

Many adsorbent materials, including activated carbon^24^ and clay^25^, have been investigated as possible adsorbents for the removal of contaminants from aqueous solutions. However, it has been demonstrated that some of these absorbents have some disadvantages. For instance, activated carbon is costly to produce and therefore unsustainable, particularly in developing nations^26^. As a result, scientists all around the world are now trying to create novel, sustainable, and reasonably priced adsorbents to remove CR and other contaminants from water^27^. Most lignocellulosic biomass wastes, such as bagasse from sugarcane, are burned or left strewn about in the open, which not only pollutes the environment but may also have an impact on global warming. Thus, the ideal strategy to manage these wastes is to valorize the biomass wastes from sugarcane bagasse and turn them into useful products like biosorbents^28^. Utilizing sugarcane bagasse waste will also encourage cleaner production and a circular green economy^29^. Bagasse from sugarcane might perhaps be processed into cellulose^30^, lignin^31^, hemicellulose^32^, and biochar^33^. These derivatives are presently being investigated as potential building blocks for the creation of various bio-composites that will be used in the treatment of wastewater.

Due to its superior qualities, including high surface area, pore structure, and high carbon content, biochar has garnered the interest of researchers studying water treatment technology^34^. The primary drawback of raw sugarcane bagasse biochar is that it has a negatively charged surface, which reduces its effectiveness, particularly when it comes to the adsorption of anionic pollutants like CR. A variety of modifications, particularly those involving metal or metal oxide impregnation, have been shown to increase the adsorption capability of biochar^35^.

Even though, it has been documented that impregnation of metals increases the adsorption capacity of sugarcane bagasse biochar, the majority of metals are hazardous, so additional treatment is required before the saturated biosorbent is disposed of in the environment. Thus, the wastewater treatment process is not only time-consuming but also potentially more expensive overall. Due to the significant interaction between contaminants and metals, metal-modified biochar also has the drawback of being difficult to regenerate^36^.

Likewise, chitosan (CS) is one of the most used biomolecules in the adsorption of various water pollutants due to its many superior properties such as biodegradability, biocompatibility and adsorption capacity^37^. The presence of reactive amino (-NH_2_) and hydroxyl (-OH) functional groups in the molecular structure of CS contributes to its ability to adsorb various pollutants^38^. These groups are considered as active adsorbent sites in wastewater treatment technologies to remove certain pollutants, such as synthetic dyes and heavy metals, through various techniques including electrostatic attraction, H and chemical interactions^39^. However, the application of CS as an adsorbent in wastewater treatment technology is still limited due to its high acid solubility, filtration capacity, poor mechanical properties, and swelling in aqueous media^40^. A very effective way to overcome these limitations and improve the physicochemical properties of CS biopolymers is chemical modification by cross-linking and/or compositing with activated carbon (AC**)**^41^.

The current research paper utilizes cost-effective sugarcane bagasse agro-residue, which has great potential and is chemically modified with chitosan to create a biocomposite with a high capacity for adsorbing dyes. Sugarcane bagasse is recognized as one of the most plentiful agro-residues^42^. Different pollutants present in diverse water sources can chemically interact with sugarcane bagasse, which consists of cellulose, hemi-cellulose, lignin, and macromolecules containing hydroxyl groups^43,44^.

In this article, we describe how to alter sugarcane bagasse biochar, which may be used again to remove CR dye from aqueous solutions. This work was smart in that it improved the sugarcane bagasse biochar’s ability to adsorb anionic dyes like CR by using chitosan biopolymer as a modifying agent. To the best of our knowledge, chitosan and biochar made from sugarcane bagasse have not been investigated as biosorbents for the adsorption of CR from aqueous solutions in bio-composites.

The objectives of this study were (i) Fabrication of chitosan impregnated sugarcane bagasse biochar (SCNC) biocomposite (ii) to investigate the physico-chemical properties of the prepared biocomposite using various instruments (iii) to investigate the effect of different parameters during the adsorption of CR like pH, dosage, initial dye concentration, time, and co-existing ions; (vi) in addition, the experimental data were fitted at different kinetic adsorption models and adsorption isotherm models to determine the mechanism of CR adsorption on the surface of the SCNC bio-composite; (v) statistical analysis of the kinetic and isotherm models using the chi-square statistic (χ^2^ ), mean square error (MSE), and the sum of squares error (SSE) and Hybrid error; (vi) comparative evaluation of dye removal efficiency (RE), feasibility, and reusability of SCNC biocomposite with other adsorbents; (vii) Elucidation of the mechanisms involved in the processes of adsorption of CR onto SCNC biocomposite. Moreover, the biocomposite was also tested in real water samples.

## Materials and methods

### Chemicals

Congo Red (C_32_H_22_N_6_Na_2_O_6_S_2_, ≥ 97% ) (Fig. S1a), chitosan (≥ 99%)(Fig. S1b) and acetic acid(≥ 99.7%) (CH_3_COOH) were all analytical reagent grades and purchased from Sigma Aldrich. All chemicals used in this present study were used without any further purification. De-ionized water was used for preparation of all chemical solutions required. CR stock solution was prepared by dissolving about one gram of dye in 1 L of deionized water.

### Preparations

#### Preparation of sugarcane bagasse (SCN) biochar

Sugarcane bagasse residues (SB) were collected from native market in Mansoura city, Egypt. Preparation of SCN was performed according to previously reported in the literature^45,46^. The SB was washed thoroughly using de-ionized water and dried in a laboratory oven at 105°C for 24 h. The dried SB was smashed and then washed with 0.5%HCl to remove all dirts, dried overnight at 105 °C, sieved to mesh size of 1–4 mm, and then put in a tabular furnace for carbonization at 600 °C for 2 h to obtain the biochar of sugarcane bagasse. Then, this residue was immersed in a solution of NaOH with the ratio of (1:3w/w). Thereafter, the obtained sample is dehydrated in oven at 110 °C and activated at 750 °C for about 2 h using muffle furnace to yield NaOH-SB activated biochar (SCN).The base acted as a dehydrating agent restraining the tar structure, thus producing a refined porosity in the resulting activated carbon. The obtained SCN biochar sample was washed with de-ionized water to remove any of remaining chemicals till neutrality of water pH. The SCN biochar particles were, then, dried overnight in an oven at 120 °C. The dried particles were pulverized and sieved. Particles of sizes ranging between 250 and 300 μm were selected for chitosan (CS) impregnation.

#### Preparation of Chitosan impregnated sugarcane bagasse biochar (SCNC) biocomposite

**Fig. 1:**
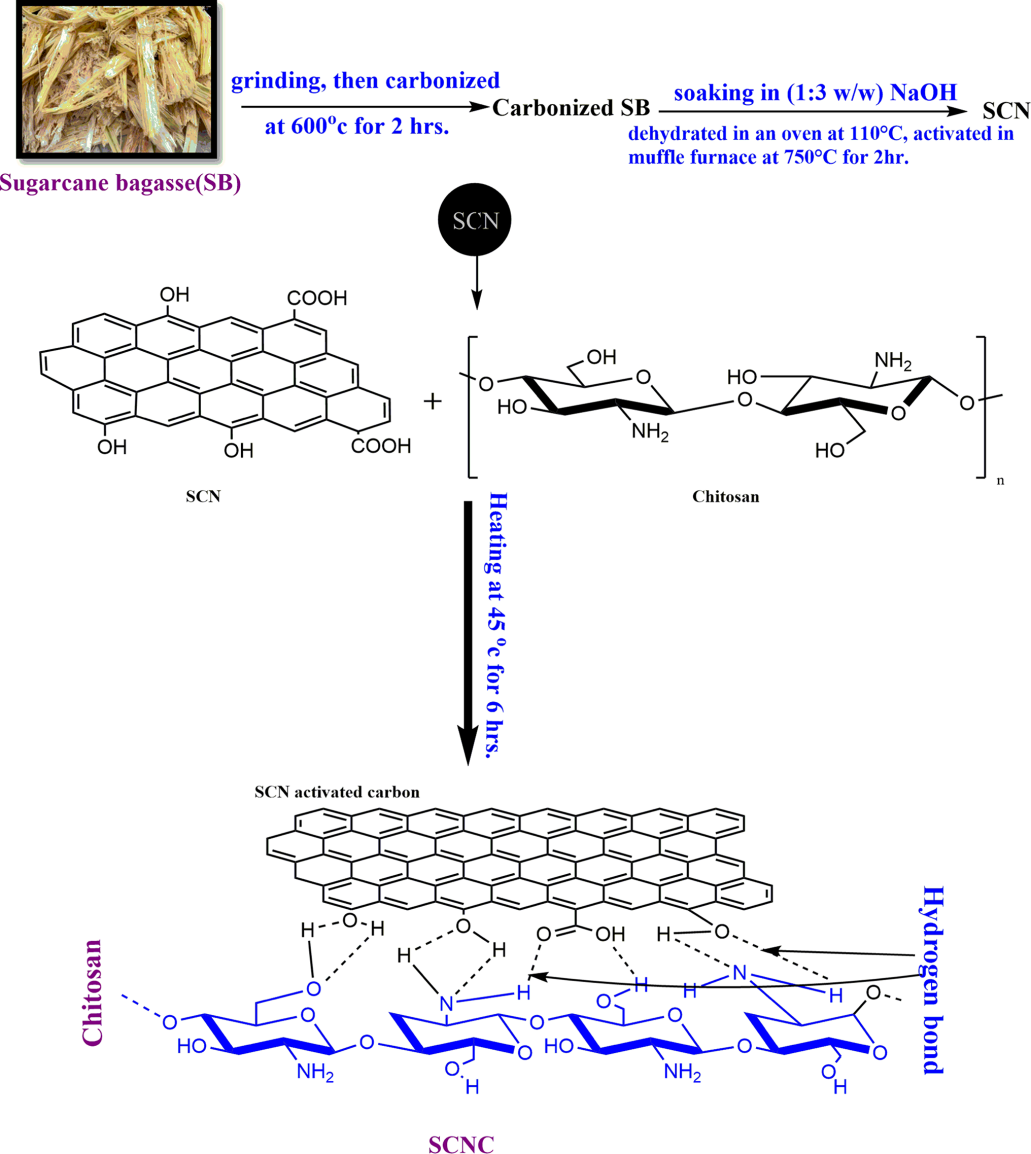
Preparation of SCNC biocomposite.

The SCNC biocomposite was prepared as previously reported^47^. For the preparation of the SCNC biocomposite, 2 g of chitosan flakes were dissolved into 100mL of acetic acid solution (2%v/v) with continuous stirring at 45°C for 6 h. After complete dissolution of chitosan, 2 g of the obtained SCN sugarcane bagasse biochar was added to the solution with vigorous stirring at 45°C for 6 h. After complete reaction, the obtained SCNC biocomposite was filtered and washed thoroughly with distilled water till neutral pH is obtained. After washing with deionized water, the prepared adsorbent was oven-dried at 110ºC overnight and then placed in a desiccator for further use. The preparation of SCNC biocomposite is schematically represented in Fig. 1.

### Characterization

Measurements of N_2_ adsorption isotherm and Brunauer, Emmett and Teller (BET) of the sample are done by nitrogen adsorption at 77 K using surface Area & Size Analyzer (QUANTACHROME – NOVA 2000 Series). The surface pH of the adsorbent was determined by shaking about 0.05 g of the adsorbent in 25mL of pre-boiled water free from CO_2_ for about 48 h; then the pH of the supernatant was determined. The pH of the point of zero charge (pH_PZC_) was determined according to previously reported work^48^. The ash content of the prepared adsorbent is determined to measure minerals present as impurities in the adsorbent which remain upon burn off of the carbonaceous portion during pyrolysis. About 0.5 g of adsorbent was put in muffle furnace for about 6 h at 700°C and then the final weight was recorded^46^. The moisture content is determined by heating 0.05 g of the adsorbent in an oven at 110^o^C overnight; then it was cooled to room temperature in a desiccator and then weighted^46^. Elemental analysis of the prepared biocomposite was performed by Perkin Elmer 2400 CHNS analyzer. Fourier transform infrared (FTIR) spectral analysis of the SCNC biocomposite and the SCNC-CR complex, was performed using Shimadzu 5800 instrument where the samples are compressed into pellets using KBr at high pressure and at 500–4000 cm^− 1^ to determine the functional groups of the prepared samples that were obtained. Surface morphology of the prepared biocomposite was determined using Scanning electron microscopy (SEM-Quanta FEG-250). The thermo-gravimetric analysis (TGA) of the sample was performed with a TGA Thermo-Gravimetric Analyzer (Shimadzu, japan) under N_2_ gas flow with a heating rate of 10 °C/min. The concentrations of CR in the supernatant aqueous solution after adsorption process were determined spectrophotometrically using a UV-Vis spectrophotometer (JENWAY 7300, Ltd., UK) double-beam spectrophotometer. The wavelength at which the maximum absorbance of CR in aqueous solution occurred was determined by scanning the light absorption curve and is equal (*λ*_max_ = 498 nm).This wavelength was used to analyze the concentrations of CR supernatant in aqueous solution after making calibration curve of CR at different known concentrations. Measurement of pH of the prepared solutions is performed using pH meter (Hi 931401, HANNA, Portugal). All chemicals that are used in the present study were weighed using electronic analytical balance. The kinetics and thermodynamic parameters were studied using water bath shaker.

### Adsorption and desorption experiments

The adsorption and desorption of CR dye from contaminated water in the experiments were analyzed by using 5mL of the solution that contains toxic dye and 5 mg of the biosorbent dose in 20 mL glass vials at different pH values, initial concentrations and contact time. All samples were acidified using 2%HNO_3_ before analysis and concentration of CR dye in the solutions is measured by UV-Vis after removal or desorption experiments at (mg/L) levels. The adsorption capacity (q_e_), removal efficiency (R,%), capacity and efficiency of the desorption were calculated from the following Eqs. 1–4, respectively^10–13^.

1 $$\:{\text{q}}_{\text{e}}=\frac{(\text{Co} -\text{Ce})\text{V}}{m}$$

2 $$\:\%\text{R}=\frac{(\text{Co}-\text{Ce})}{\text{Ci}} \times 100$$

3 $$\:{\text{q}}_{\text{d}}=\frac{\text{C}\text{d}\text{V}}{m}$$

4 $$\text{Desorption}\%=\frac{\text{qd}}{\text{qe}} \times 100$$

where, q_e_ represents the sorption capacity$\:(\text{m}\text{g}.{\text{g}}^{-1})$, *C*_*o*_ is the initial concentration of CR dye solution (mg/L), *C*_*e*_ is concentration of dye at equilibrium (mg/L) after adsorption, *V* is the CR dye solution’s volume (L), and *m* is the adsorbent mass in g. %R, is the removal percentage of dye, q_d_ is the desorption capacity expressed in (mgg^−1^), *C*_*d*_ is the concentration of dye in the eluent in (mg/L) after the adsorption experiment and %De is the desorption efficiency.

### Batch experiments

Batch experiments were executed to attain the optimum conditions that lead to the highest adsorption efficiency. 0.05 g of SCNC biocomposite as initial mass of (except for adsorbent dose effects study, mass was varied between (0.01 g to 0.08 g) is added to 25mL of 100 mg.L^− 1^ and 150mg.L^− 1^ of dye solution respectively, pH was examined from 3 to 12 by addition of 0.1 M NaOH and/or 0.1 M HCl. Firstly, the temperature was adjusted to 25°C, and thereafter differentiated to 35°C, 45°C and 60°C to study temperature effect. Initial dye concentration is ranged between 50-550mg.L^− 1^. Contact time was first set to 6 h and then extended between 1 and 24 h to estimate the contribution of contact time to adsorption behavior. Concentrations of CR ions in each experiment were measured by UV-Vis spectrophotometry. Then, adsorption capacity (q_e)_ and efficiency of CR removal (R, %) were estimated by Eqs. (1) and (2). Influence of initial dye concentration was performed by weighting 0.025 g of the adsorbent in 100mL Erlenmeyer flask containing different concentrations of CR and then shaken in orbital shaker at 160 rpm till the equilibrium is reached. To study the effect of pH on the adsorption of CR using the prepared adsorbent, 150 mg/L of the dye solution is prepared at different pH (3.0–12.0), with 0.05 g of the adsorbent for 400 min. At optimum condition of 150 mg/L dye concentration and 0.025 g dose of adsorbent at pH 3.0 for SCNC and pH 5.5 for SCN and at room temperature, the effect of contact time is evaluated and then adsorption kinetic parameters are calculated.

The influence of adsorbent dosage on the elimination of CR is determined by varying the adsorbent dose between 0.01 and 0.08 g at initial dye concentration 150 mg/L in 25mL in an Erlenmeyer flask at room temperature. After the adsorption process is totally complete the residual supernatant of CR is determined using UV-Vis spectrophotometer, at λ_max_ = 498 nm and maximum uptake (adsorption capacity) q_e_ in (mg.g^− 1^) and percentage of sorption (R %) of CR by the adsorbent were calculated from the Eqs. 1 and 2. The thermodynamic parameters were investigated as 200 mg.L^− 1^ initial concentration of CR was subjected to adsorption at different temperatures (25-60°C) to study the effect of temperature on the sorption capacity.

### Desorption experiments

The desorption efficiency of CR was executed after reaching adsorption equilibrium state by using different desorbing agents viz. absolute ethanol, nitric acid, hydrochloric acid, sodium hydroxide, sodium-bicarbonate with concentrations between (0.01 to 0.1 M). Briefly, 0.2 g of CR loaded SCNC was mixed with 50mL of desorbing agent within a pH range of 2.0–12.0 at 298 K. Then the desorbed CR concentration was analyzed spectrophotometrically after the suspension was stirred for a fixed time. The desorption efficiency of CR can be calculated using Eq. (4).

### Effect of co-existing ions

Textile industrial effluents are a complex mixture of numerous ions (anions and cations), dyes, heavy metals, and solids (dissolved and suspended solids). Textile industrial effluents also have elevated percent of biochemical and chemical oxygen demand, colour intensity, pH, and total organic carbon. These chemicals and solids could initiate interfering and/or masking effect during the process of adsorption. Accordingly, bicarbonate (HCO_3_^−^), carbonate (CO_3_^2−^), nitrate (NO_3_^−^), phosphate (PO_4_^3−^), and sulfate (SO_4_^2−^) ions were considered to evaluate the effects of co-existing ions on CR dye adsorption using SCNC biocomposite in the present study. For this purpose, four concentrations (1, 5, 10, and 50 mgL^− 1^) of each ions were prepared by dissolving the necessary amounts of sodium bicarbonate, sodium carbonate, potassium nitrate, potassium phosphate, and sodium sulfate as background electrolytes and, later, 50 mgL^− 1^ CR were prepared using these ionic solutions. Finally, the adsorption experiments using these co-existing ions containing CR solutions were used to evaluate the effects of co-existing ions on dye adsorption using SCNC biocomposite.

### Sample analysis

Several samples of surface natural water were collected from the Nile River (Damietta branch, Egypt) and seawater samples (Alexandria City, Egypt). Tap water samples were collected (Mansoura University, Egypt). All samples of water were filtered by a sintered glass G4 filter. All the selected samples were acidified with concentrated nitric acid to pH ∼2 and then preserved in polyethylene vessels for further use^46^. The organic matter was digested before the separation process; 0.5–1.0 g of K_2_S_2_O_8_ was added to one liter of the selected water sample and the mixture was heated for 30 min at 95 °C^46^. After cooling to room temperature, about 80 mg of SCNC sample was added to a group of transparent stoppered bottles that contain different concentrations (0.0, 50 and 100 ppm) of CR dye at 25°C and optimum conditions of pH = 3. The stoppered bottles were shaken at 150 rpm on an equilibrated shaker for 4 h in the presence of sunlight; then filtered. Another 80 mg of SCNC was added to the filtrate and the pH was adjusted again. The sample was stirred for 15 min again and after that, filtered. Both filtrates were gathered and then collected. The residual concentrations of CR dye were measured spectrophotometrically.

## Results and discussion

### Materials’ design and physicochemical studies

Chitosan impregnated sugarcane bagasse biochar (SCNC) has been synthesized in two step co-precipitation technique. In the first step, sugarcane bagasse biochar was synthesized and modified with NaOH. In the second step, chitosan solution was impregnated to sugarcane bagasse biochar to yield sugarcane bagasse biochar impregnated with chitosan (SCNC) biocomposite .

Both SCN and SCNC biocomposite are black in color. A change in the nature of both SCN and SCNC biocomposite can be obviously noticed form Fig.S2(a, b). The prepared biochar SCN is very fine powder with low density and lighter than SCNC biocomposite ; but the newly prepared SCNC biocomposite is hard, black and tends to be more granular than the SCN sample. The solubility of SCNC biocomposite in water was tested by placing a 1.00 g of SCNC biocomposite sample in 50.0mL water. The suspension was stirred for 3.0 h, and subsequently the resulting solid was collected through filtration, dried and weighed. There was a negligible loss of mass overall that was noticed.

#### Moisture and ash content

As illustrated in Table 1, the SCNC biocomposite showed high moisture content (12.58%) which gives an indication of having high surface area and plentiful active sites available on the surface of the prepared adsorbent and the presence of hydrophilic functional group on the hydrophobic composite surface. The ash content was very low ∼2.97%. This decrement may be attributed to the increase in the carbon yield; and this can be determined and confirmed from the elemental analysis which can approve the high carbon content which reaches (67.53%) compared to other elements of hydrogen and nitrogen as shown in Table 1.

**Table 1:** Physicochemical properties of SCNC biocomposite.

Biosorbent	pH of supernatant	pH_PZC_	Ash %	Moisture %	S_BET_ (m^2^/g)	Total pore volume (cm^3^/g)	Mean pore diameter $({\overline{r}})$nm	Elemental analysis		
SCN	10.46	9.86	4.67	29.62	697.369			C (%)	H (%)	*N* (%)
								77.29	0.826	0
SCNC	6.17	6.43	2.97	12.58	481.66	0.3210	2.66	C (%)	H (%)	N (%)
								67.53	3.26	2.743

#### BET surface area

**Fig. 2:**
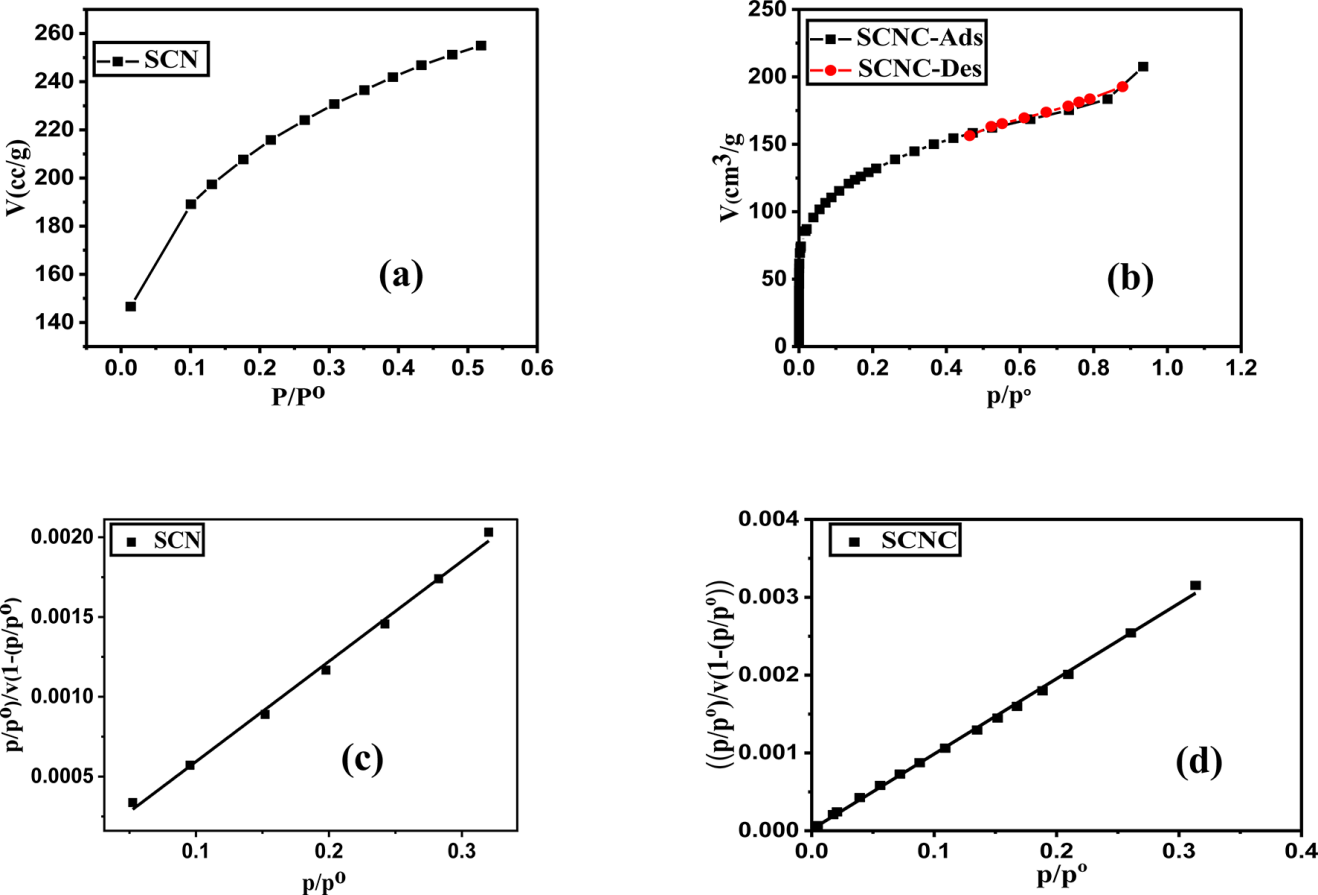
(**a**,** b**) Nitrogen adsorption- desorption isotherms at 77 K, (**c**,** d**) Linear BET plots of nitrogen adsorption isotherms at 77 K for SCN&SCNC biocomposite.

Adsorption capacities of adsorption strategy are mainly dependent on both carbons’ porosity and their surface area^49^. The textural features of solids are conventionally obtained from the nitrogen adsorption at (77 K), Fig. 2(a&b) and then the data of adsorption are analyzed by the application of the BET equation, Fig. 2(c&d)^50^. It was discovered that the SCNC biocomposite adsorbed nitrogen relatively quickly, with equilibrium being reached in less than 25 min demonstrating that active diffusion, which is portrayed in ultrafine pores, is not what controls adsorption, while also referring to the accessibility of the entire pore structure to the nitrogen molecules.Fig. The SCNC biocomposite’s surface area values were determined using linear BET plots of N_2_ adsorption at 77 K. According to the results, Fig. 2; Table 1, the S_BET_ value of SCN biochar is 697.369 m^2^/g, whereas that of SCNC is 481.66 m^2^/g. The impregnation of chitosan into SCN biochar may be responsible for this reduction in surface area. After chitosan impregnation, the specific surface area may have decreased as a result of the SCNC biocomposite holes being covered by fixed chitosan moieties, which reduced the adsorption of N_2_ molecules. The decrease in surface area of the functionalized SCNC biocomposite would suggest that the coordination of CR dye with the functional groups onto SCNC is the primary mechanism by which the adsorption process occurs. Moreover, The mean pore diameter of SCNC biocomposite (26.6 Å) is greater than the length of CR (18.3 Å) allowing the easy diffusion of CR molecules into the pores of SCNC biocomposite.

### Characterization

#### SEM

**Fig. 3:**
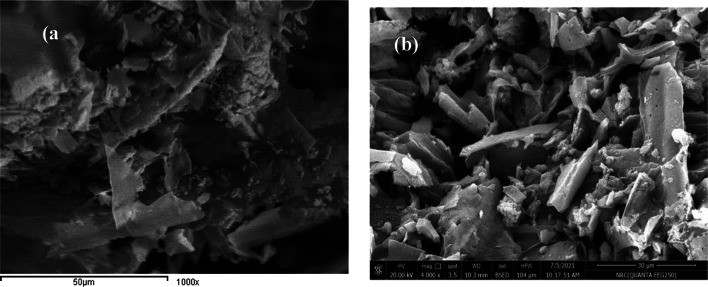
(**a**) SEM micrograph of SCN biochar without chitosan, (**b**) SCNC biocomposite with chitosan.

Surface morphological properties of the prepared SCN biochar and CS impregnated biochar SCNC are represented in Fig. 3(a&b). A large difference between the SCN biochar and CS impregnated biochar )SCNC( before and after impregnation of biochar sample with chitosan can be noticed. These images explain how CS impregnation affected the surface morphology of the SCN biochar; where the SCN biochar sample before impregnation is more regular with smooth nature. After CS impregnation, the surface of SCNC biocomposite became more irregular indicating that an interaction between SCN biochar and CS had occurred.

#### FTIR

The FTIR spectral analysis of SCNC biocomposite before and after adsorption of CR can be used to study the interaction between adsorbent and dye molecules.

##### FTIR spectra of SCNC biocomposite before CR adsorption

**Fig. 4:**
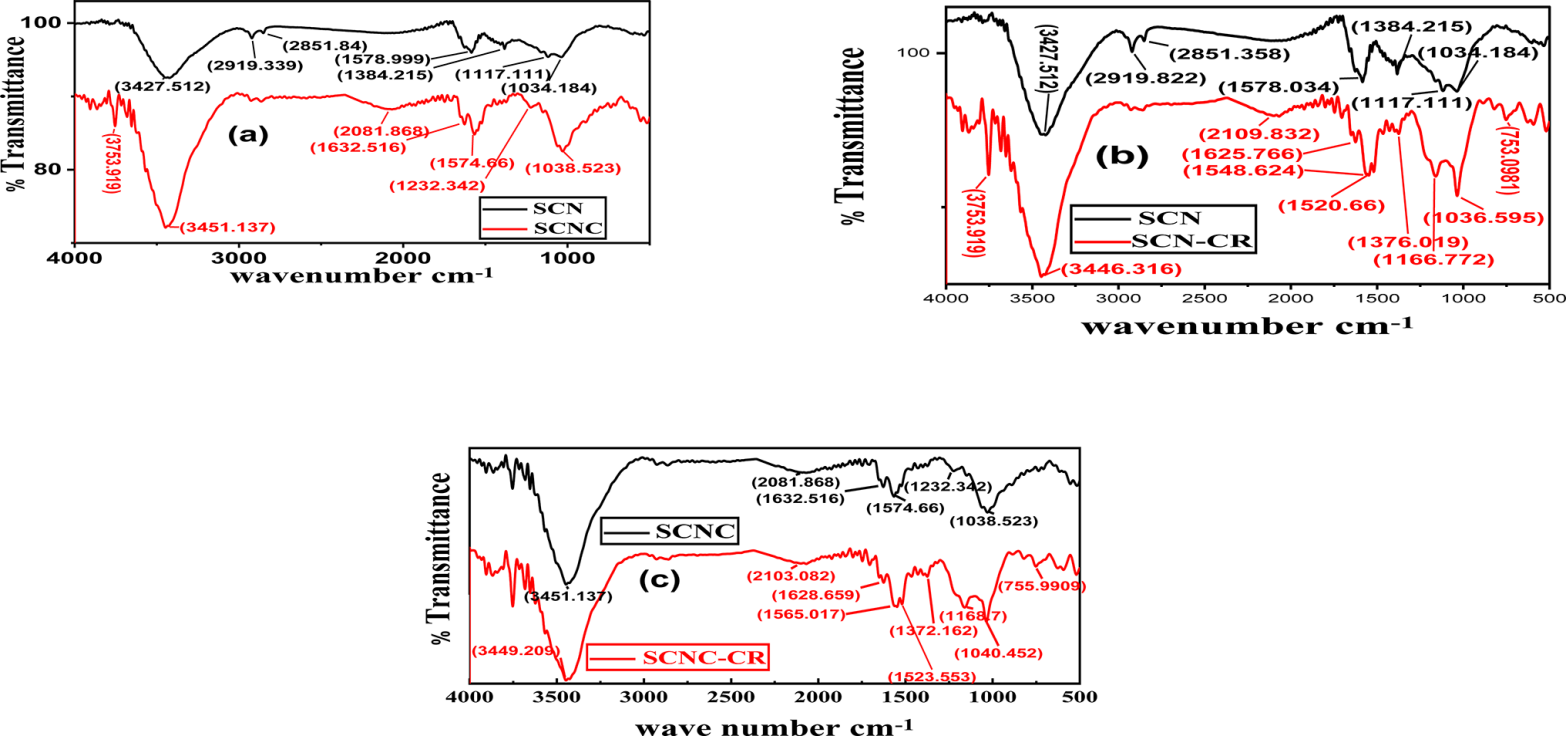
FTIR of (**a**) SCN and SCNC biocomposite, (**b**) SCN-CR, and (**c**) SCNC-CR.

The broadband around 3449–345 cm^− 1^ represented in Fig. 4(a), is a distinguishing of the -OH groups stretching vibration (from carboxyls, phenols or alcohols), N-H groups and adsorbed water in the SCNC biocomposite. Small intense band at 2081–2103 cm^− 1^, was ascribed to ʋ(C$\:\equiv\:$C) vibration in alkyne groups of the prepared biochar sample. The bands between 1628 and 1632 cm^−1^ are owing to C = O stretching mode of quinine and ketones. This π bond containing organic group might create π-π stacking with the delocalized π bonds of aromatic ring of CR molecules, resulting in enhancement of adsorption efficiency. The bands between 1574 and 1565 cm^−1^ is owing to π bond containing functional group C = C vibrations in aromatic rings. In Fig. 4(b), the beak at 1232 cm^−1^ is owing to stretching mode of C = O in carboxylic group. Another band present around 1232 cm^− 1^ would be related to C–O stretching of ether group and O-C-O stretching of COOH^51^.

##### FTIR spectra of SCNC biocomposite after CR adsorption

After adsorption of CR onto SCNC biocomposite, Fig. 4(c), new three beaks appeared which indicate the CR adsorption; the beak at 1523 cm^− 1^ is due to secondary amide N-H bending. The band at 1372 cm^− 1^ is due to aromatic amine C-N stretching mode and also due to N-N azo-compound stretching mode of CR. Another two absorption bands appearing around 1168 cm^− 1^ and 755–822 cm^− 1^ could be ascribed to the stretching of S$\:=$O in SO_3_ and N-H in CR dye that confirm the adsorption of CR dye onto the SCNC biocomposite. This may be because of the electrostatic reaction between (-SO_3_^−^) sulfonated group present in CR dye and protonated amino NH^3+^ groups^52^. Hence, it could be concluded that the adsorption of CR dye mechanism was by variety of anionic moieties of dye molecule and protonated amino (NH^3+^) groups of SCNC biocomposite .

#### TGA

**Fig. 5:**
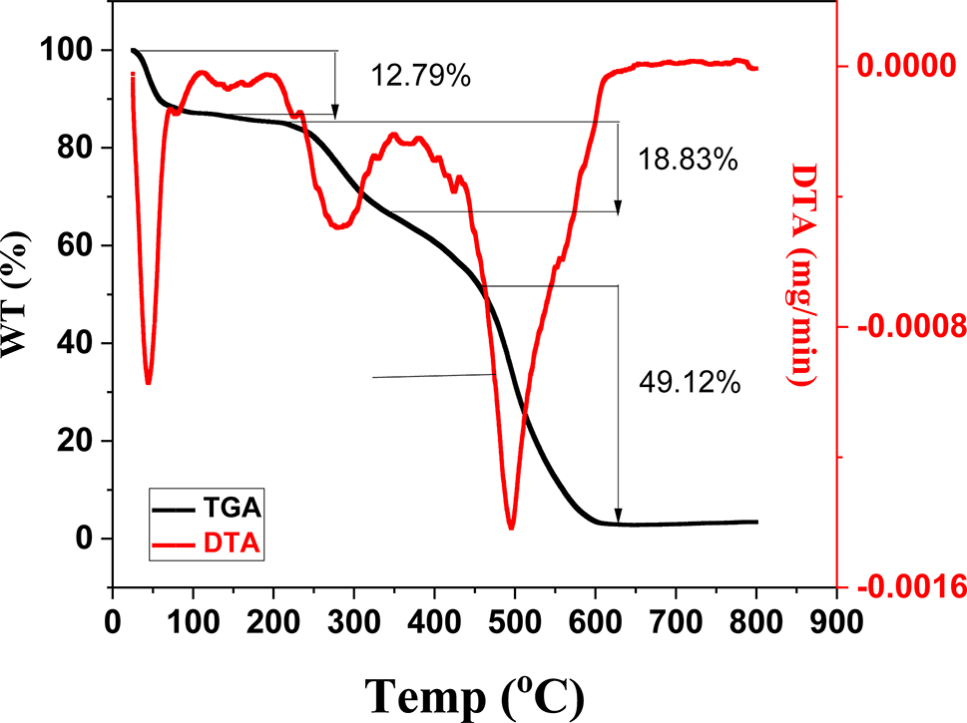
Thermal analysis of SCNC biocomposite .

The SCNC biocomposite is thermally analyzed in the temperature range between 25-800°C to get information about the thermal stability against temperature by recording weight loss by increasing temperature up to 800°C. As shown in Fig. 5, SCNC biocomposite exhibits three decomposition steps. From Fig. 5, there is weight loss at low rate, from temperature (25-500°C) to record weigh loss between (12.79 − 18.83%). From temperature 500 to 800°C, there is regular decomposition with weigh loss to be 49.12%.The complete degradation above 500°C with (49.12%) degree gives an indication on the high stability of this adsorbent against temperature because complete degradation didn’t occur in the first step but occur above 500°C.

### Adsorption studies

#### Surface pH and point of zero charge (pHPZC)

**Fig. 6:**
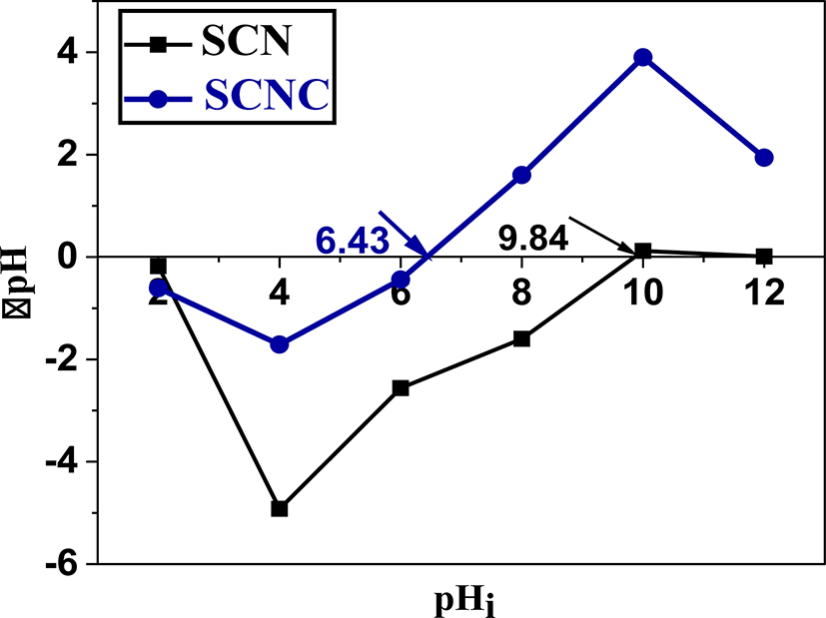
pH_PZC_ of SCN and SCNC biocomposite.

The surface pH and the point of zero charge (pH_PZC_) are two important parameters in determination of the functionalities present on the carbon surface. In case of the present adsorbent, (as shown in Table 1) the surface pH equal (6.17) which is attributed to the basic functional groups on the sample surface. The pH_PZC_ describes the condition at which the density of electrical charge on a surface is equal to zero or it is the pH at which the adsorbent surface has positive and negative charges at equal amount, resulting in net zero charge^53^. Generally, at pH < pH_PZC_ the surface of SCNC biocomposite is positively charged and surface sites are protonated, however when the pH > pH_PZC_, the surface of SCNC is negatively charged. This charge on the SCNC surface is one of the parameters that illustrates the adsorption characteristics^54^. In our study, the SCNC surface is positively charged at which pH_PZC_ (6.43) is slightly higher than surface pH (6.17) as presented in Fig. 6.

#### Influence of pH

**Fig. 7:**
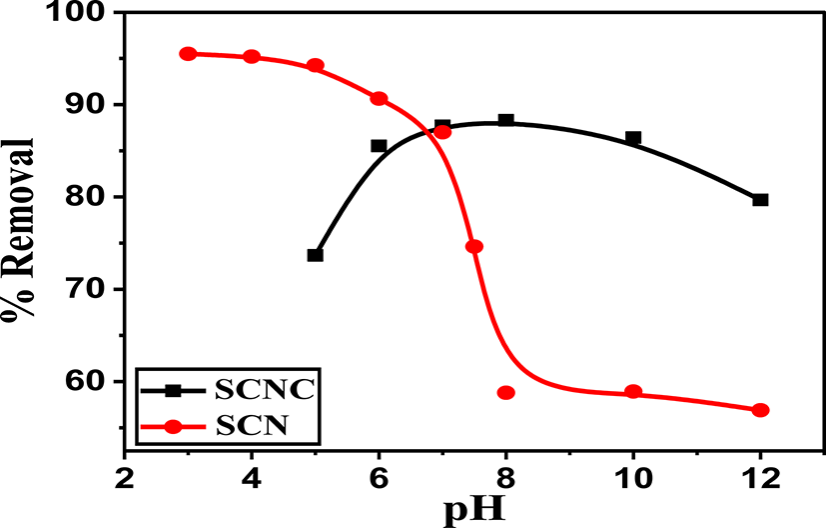
Effect of pH on adsorption of CR onto SCNC biocomposite and SCN.

The elimination of CR dye basically depends on the pH of the dye solution, because it influences the surface polarity of the adsorbent, ionic mobility and chemical properties of the CR dye solution^55^. The influence of pH of sample solution on the removal of CR using the SCNC biocomposite is presented in Fig. 7. When solution pH > pHpzc, the surface of the adsorbents become negatively charged and favors the removal of cationic dye but when at solution pH < pHpzc, the adsorbent surface becomes positively charged and then adsorbs the anionic dye such as CR^56^. As shown in Fig. 7, the removal of CR decreases from (98.0%) at pH = 3.0 to be (80.0%) at pH = 4 for SCNC biocomposite and decreases from (85.0%) at pH = 5 to be (79.0%) at pH = 12 for SCN. So, the adsorption efficiency decreases with increasing of the solution pH. As the pH of the CR solution increased, a proportional decrease in adsorption took place due to the successive deprotonation of positive charged groups on the adsorbent and electrostatic repulsion between negatively charged sites on the adsorbent and dye anions. There was also competition between OH^−^ (at high pH) and dye anions for the positively charged adsorption sites.

#### Influence of adsorbent dosage

**Fig. 8:**
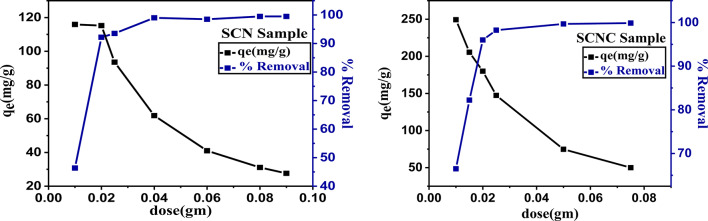
Effect of dose of SCN and SCNC biocomposite on CR removal.

One of the crucial variables is the adsorbent dosage, which indicates the adsorbent’s capacity for a particular initial concentration of the adsorbate.The removal of CR dye from an aqueous solution can essentially be influenced by varying dosages of the produced biocomposite. As illustrated in Fig. 8, when the adsorbent dosage is increased from 0.01 to 0.08 g, resulting in a decrease in the adsorption capacity, the removal efficiency of CR dye increases from (65.0%) up to reach approximately (100.0%) in case of SCNC biocomposite and increases from (45.0%) up to reach about (100.0%) in case of SCN. Due to the adsorbent’s increased availability of active sites towards CR, the CR removal.

increases quickly as the adsorbent dose increases.The dye removal efficiency remains.

constant at a given adsorbent dosage and is unaffected by increasing the adsorbent dosage^57,58^. The dispersion of SCNC particles in the bulk solution is thought to be better at low adsorbent dosages; this means that all of the active sites on the adsorbent surface are fully exposed, which may hasten the approachability of pollutant molecules to a significant number of the adsorbent active sites. As a result, a high removal capacity is achieved by the adsorption on the surface active sites reaching a saturated point. But when the amount of adsorbent is increased, more of the lower-energy active sites become occupied and the accessible high-energy adsorbent active sites become less accessible, which slows down the adsorption process.

#### Influence of initial CR concentration

**Fig. 9:**
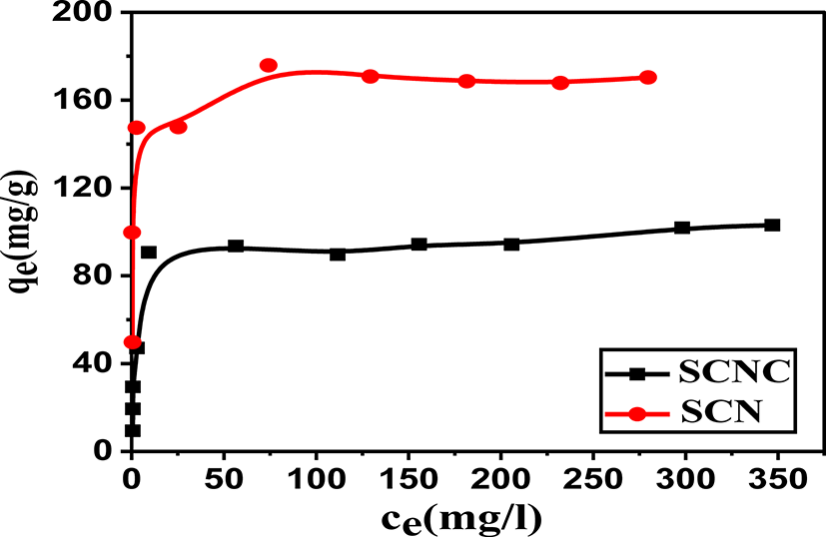
Effect of CR concentration on the adsorption efficiency of SCN and SCNC biocomposite samples.

The results of the experiments varying the initial concentrations of the studied pollutant(2.5–300 mg/ L) over the SCNC biocomposite are illustrated in Fig. 9. When the initial concentration of CR was increased, the adsorption capacities increased until they reached a maximum level at 170 mgL^− 1^, Fig. 9. The increase in the loading capacities of SCNC biocomposite with increasing CR concentration is due to the interaction between CR and the SCNC biocomposite which provides the vital driving force to defeat the resistances to the mass transfer of CR between the aqueous solution and the SCNC biocomposite. Additionally, the following explanations could account for this phenomenon: at low CR/SCNC ratios, CR adsorption occurs on the high-energy sites; however, as the ratio rises, the higher energy sites become saturated and adsorption moves on to the lower energy sites, resulting in a decline in adsorption efficiency (similar findings have been reported in the literature previously)^59–62^.

#### Adsorption isotherms

The adsorption isotherm illustrates the retention or release of a substance from the aqueous phase to the solid phase at a constant temperature, a concept that is widely recognized. It is essential to comprehend the characteristics of the adsorption surface and the interaction mechanisms between the adsorbate and the adsorbent surface. This study examines two isotherm models, specifically the Langmuir and Freundlich isotherm models, to identify the most suitable equilibrium curves. The Langmuir isotherm, as described in Eq. 5, asserts that once an adsorbate occupies a site, further adsorption at that location ceases, resulting in a distinct plateau on the curve, with no side commerce or steric interference among the adsorbed molecules. Conversely, the Freundlich isotherm, represented in Eq. 6, is regarded as an empirical model that accounts for interactions among adsorbed molecules (multilayer adsorption) on heterogeneous surfaces characterized by a uniform energy distribution. This model also indicates that the concentration of the adsorbate on the adsorbent surface will increase with rising adsorbate concentration in the solution, without reaching saturation.

In this work, the Langmuir and Freundlich isotherms were used to assess the experimental data^63–66^. Their linear forms are given by Eqs. (5, 6), respectively.

5 $$\:\frac{{c}_{e}}{{q}_{e}}=\frac{1}{bq}+\frac{{c}_{e}}{q}$$

6 $$\:\text{log}{q}_{e}=\text{log}{K}_{F}+\frac{1}{n}\text{log}{C}_{e}$$

where q_m_ represents the system’s maximum adsorption capacity (mg g^− 1^), and q_e_ represents the quantity of CR dye adsorbed at equilibrium onto the surface of the SCNC biocomposite (mg g^− 1^), C_e_ is the concentration of adsorbate at equilibrium (mgL^− 1^). K_L_ is constant for Langmuir (Lmg^− 1^), n is adsorption intensity, b is the adsorption affinity constant (Lmg^− 1^), and K_f_ is the Freundlich constant ((mg g^− 1^)(Lmg^− 1^ )^1/n^), linked to the adsorption capacity.

In order to explore the well-fitting kinetic and isotherm models and to reduce the error distribution between the computed values from theoretical model correlations and experimental data, a variety of error functions were used. The chi-square statistic (χ^*2*^), mean square error (MSE), and sum of squares error (SSE) and hybrid error were the four error functions used^67^.

**Fig. 10:**
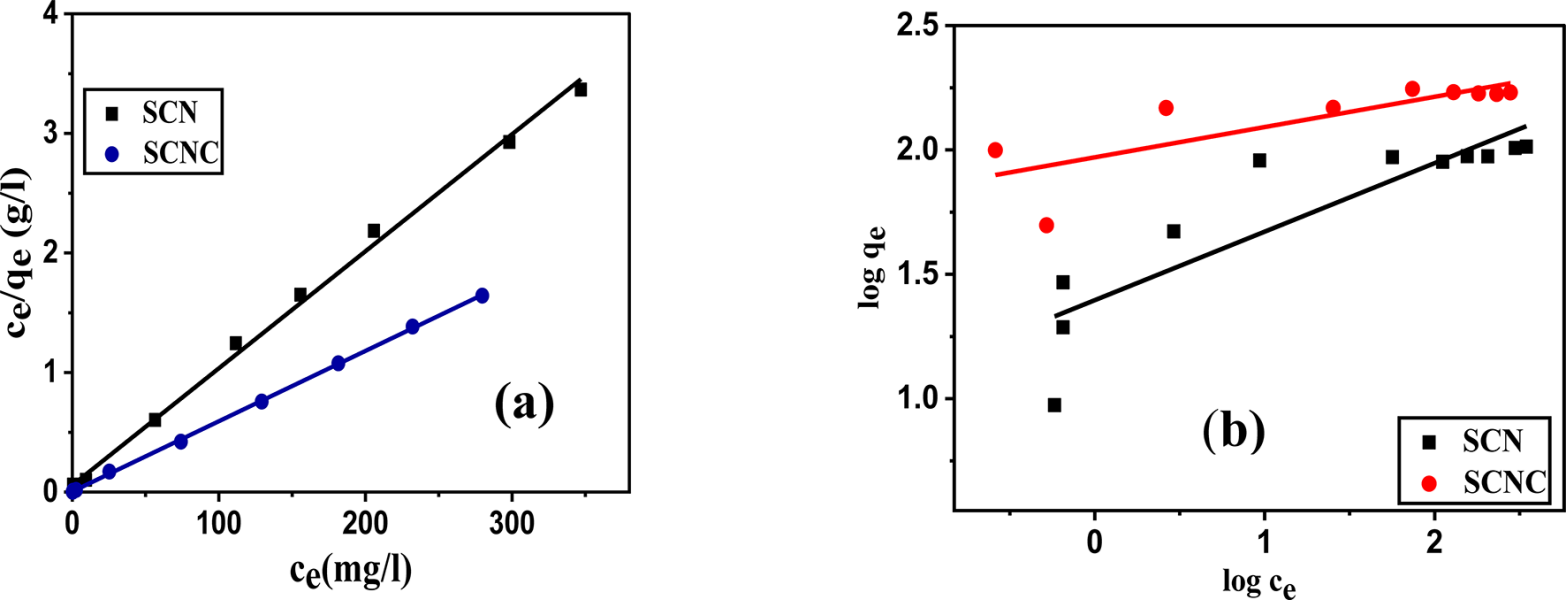
(**a**) Langmuir isotherm, and (**b**) Freundlich isotherm model.

The linear fitted curves are shown in Fig. 10, and the data for each isotherm model of CR adsorption on the SCNC biocomposite are reported in Table 2.

Table 2 provides the fitted parameter values for the adsorption isotherm models. Given the higher correlation coefficient (R^2^ ≥ 0.999) and lower χ^2^, MSE, SSE and hybrid error values, the equilibrium CR adsorption isotherms for a single-dye system fit the Langmuir isotherm model.

In addition, the characteristics of Langmuir isotherm R_L_, the separation factor, and a dimensionless constant is defined as

7 $${R}_{L}=\frac{1}{1+{K}_{L}{C}_{O}}$$

When the value of R_L_ is between 0 and 1, the adsorption process is favorable. It is unfavorable when R_L_ is more than 1, irreversible when R_L_ is zero, and linear when R_L_ is 1. By analyzing the generated data, it can be shown that the R_L_ values are within 0 and 1, suggesting highly favorable adsorption^68^.

**Table 2:** Langmuir and Freundlich isotherm parameters.

Model	Parameter	SCNC
Langmuir	q_m_ (mg/g)	170.06
	b (L\mg)	1.32
	R_L_	0.0016
	R^2^	0.999
	χ^2^	120.41
	SSE	20477.57
	MSE	2275.28
	Hybrid error	120.41
Freundlich	K	93.32
	1/n	0.121
	R^2^	0.584
	χ^2^	157.614
	SSE	29081.50
	MSE	3231.27
	Hybrid error	5770.26

#### Influence of contact time and adsorption kinetics studies

**Fig. 11:**
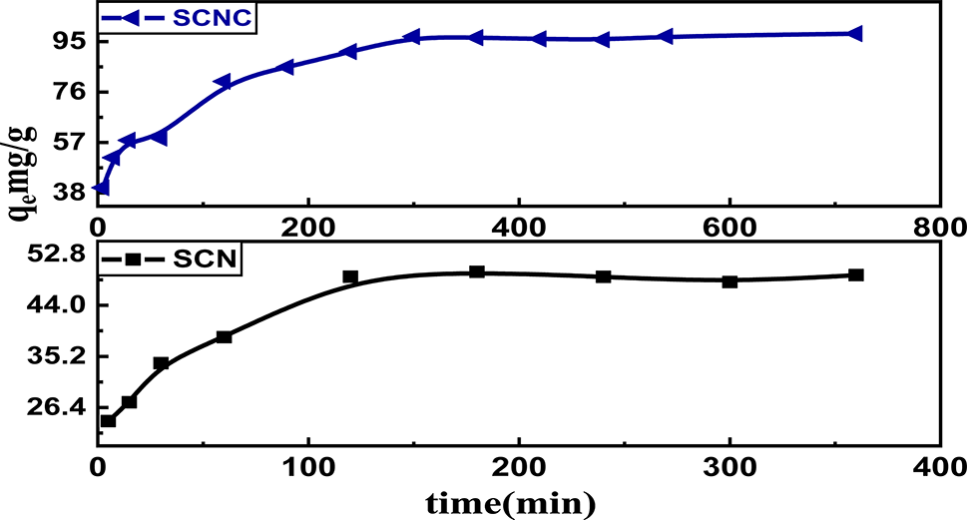
Effect of contact time on SCN and SCNC.

The kinetics of CR adsorption onto the SCNC biocomposite were assessed, focusing on the effect of contact time (t in min) on the adsorption capacity (qt in mg/g). As illustrated in Fig. 11, the adsorption capacity increases over time, which can be divided into three distinct phases. The initial phase, occurring within the first 30 min, is marked by rapid adsorption, primarily attributed to the abundance of available sites on the SCNC biocomposite surface. As these active sites become occupied, the second phase commences, characterized by a slower adsorption rate that spans from 30 to 200 min. During this period, CR molecules attempt to diffuse into the pores and are gradually adsorbed within the internal pores until equilibrium is reached, which occurs after 200 min. The final phase, where a plateau is observed, signifies the saturation of the biosorbent’s reactive groups with CR molecules^69^.

Several kinetic models were evaluated in order to examine the rate, mechanism, and rate controlling step of the adsorption behavior of CR onto the SCNC biocomposite .

In the current work, test data were utilized to execute the pseudo-first order(PFO), pseudo-second order(PSO), intra-particle diffusion (ID) and Boyd ‘s models^70–73^. The following Eqs. (8–11), respectively, provide their non-linear versions:

**PFO Eq. **

8 $$\:\text{log}({q}_{e}-{q}_{t})=\text{log}{q}_{e}-\frac{{k}_{1}}{2.303}t$$

**PSO equation**:

9 $$\:\frac{t}{{q}_{t}}=\frac{1}{{k}_{2\:}{q}_{e}^{2}}+\frac{1}{{q}_{e}}\:t$$

**Weber-Morris Intra-Particle Diffusion**

10 $$\:{q}_{t}={k}_{int\:}{t}^{0.5}+c\:\:\:\:\:\:\:\:$$

**Boyd’s model equations** 

11 $$\:F\left(t\right)=1-\frac{6}{{\pi\:}^{2}}{\sum\:}_{n=1\:}^{\infty\:}\frac{1}{{\:\:n}^{2}}\text{\:exp}(-{n}^{2}Bt)$$

where qe and qt represent the adsorption capacity (mgg^− 1^) at equilibrium and contact time t, the rate constants for the pseudo-first and pseudo-second order equations were k_1_ (min^− 1^) and k_2_ (gmg^− 1^ min^− 1^), respectively, whereas k_int_ was the rate constant for intra-particle diffusion (mg g^− 1^ min^− 1^) and C represents the thickness of the boundary layer, $\:F$ is the fractional of equilibrium at different times (t), and $\:B\:\left(t\right)$ is the mathematical function of$\:\:F$. *n* is an integer which defines the infinite series solution and $\:F$is the equilibrium fractional attainment at time $\:t.$

**Fig. 12:**
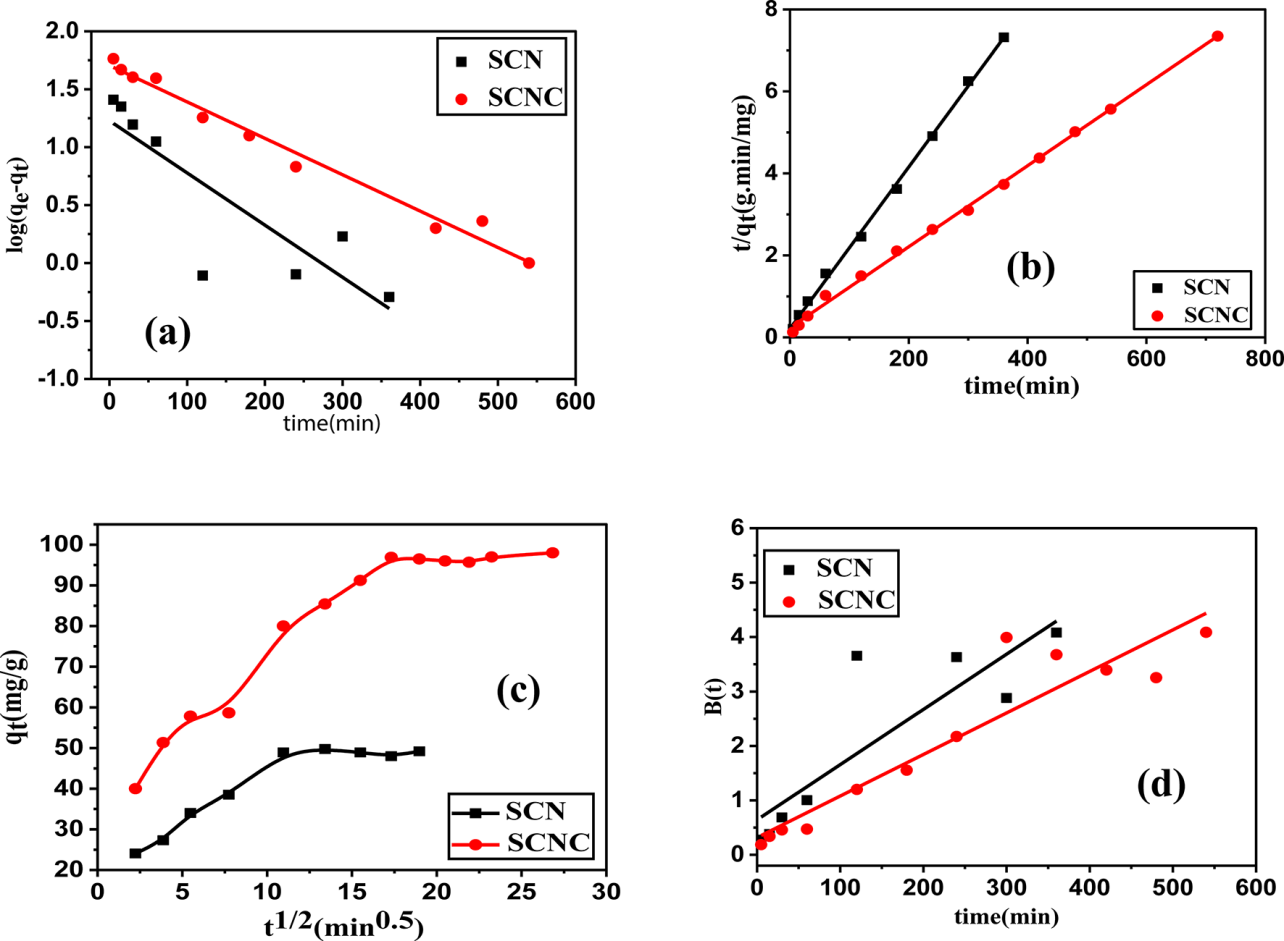
(a) PFO kinetic model, (b) PSO kinetic model, (c) Intra particle diffusion (d) Boyed’s model.

The various kinetic parameters employed in this investigation are represented in Fig. 12(a-d) while Table 3 enlists the values of these parameters. When compared to the PFO parameter, the correlation coefficient value (R^2^) for the PSO parameter was highest, as shown in Table 3. The calculated qe (mgg^− 1^) for SCN and SCNC biocomposite was determined to be 50.68 and 101.21, respectively, as can be seen from the PSO order. Based on the highest correlation coefficient R^2^(0.997 and 0.998 for SCN and SCNC biocomposite, respectively), the PSO kinetic model seems to be the best suitable model for explaining the adsorption of CR dye onto SCNC biocomposite. Furthermore, the χ^2^, SSE, MSE and hybrid error values associated with the PSO are considerably lower than those of the PFO, indicating that the PFO does not adequately align with the experimental data for CR.

Figure 12c represents the multilinear adsorption of CR onto SCNC biocomposite. As it can be noticed, two or more stages were involved in the adsorption process. The first linear step was caused by the adsorbate external diffusion onto the adsorbent, while the second linear step was caused by the delayed intra-particle adsorbate diffusion. The final step included a demonstration of establishing equilibrium. The divergence from the origin in the first and last phases of adsorption is caused by changes in mass transfer. The thickness of the boundary layer and the presence of multi-linearity indicate that the adsorption process may also involve a process such as external diffusion, film diffusion, or surface adsorption. This implies that there were other rate-controlling steps involved in the adsorption process besides intra-particle diffusion. Furthermore, the Boyd’s graph in Fig. 12(d) did not pass through the origin, suggesting that film diffusion is the rate-limiting adsorption process for CR adsorption on SCN and SCNC biocomposite.

**Table 3:** Kinetic parameters for the adsorption of CR adsorption.

Model	Parameter	SCNC
PFO	q_eexp_(mg/g)	98
	q_1_ (mg/g)	50.11
	k_1_ (1/min)	0.0072
	$\:{R}_{1}^{2}$	0.979
	*χ*^2^	340.905
	SSE	17082.792
	MSE	1314.06
	Hybrid error	1968.11
PSO	q_2_ (mg/g)	101.21
	k_2_ [g/(mg.min)]	4.18 × 10^− 4^
	$\:{R}_{2}^{2}$	0.998
	χ^2^	107.22
	SSE	10852.68
	MSE	837.33
	Hybrid error	1670.50
IPD	k_int_ [mg/(g. min^1/2^)]	2.25
	_C_	55.70
	$\:{R}_{2}^{2}$	0.941
Boyd’s equation	Intercept	0.316
	R^2^	0.869

#### Effect of temperature and thermodynamic studies

**Fig. 13:**
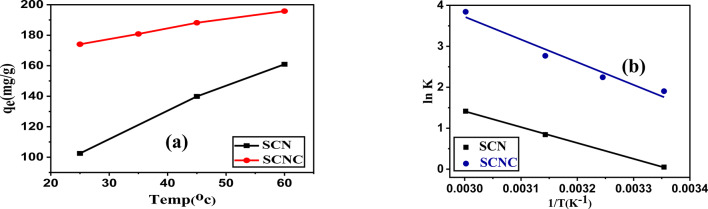
(**a**) Effect of temperature and (**b**) Vant Hoff equation.

In addition, the equilibrium adsorption capacity of CR onto the preferred adsorbent, SCNC biocomposite, was examined at elevated temperatures ranging from 20 to 60.0 °C at a pH of 4.0 (Fig. 13a). The results indicated that increasing the temperature of the CR solutions from 25 °C to 60 °C resulted in an enhancement of the removal efficiency of SCNC biocomposite from 87 to 98%. This observation suggests that the adsorption of organic pollutants onto the active sites of the SCNC biocomposite sample is an endothermic process, which may be attributed to the increased availability of active sites on the adsorbent, as well as the expansion and activation of the adsorbent surface at higher temperatures. Additionally, this phenomenon could be linked to the improved mobility of organic pollutants from the bulk solution to the adsorbent surface, thereby facilitating access to the active sites.

To gain a deeper understanding of the influence of rising temperature on the adsorption of CR onto the active sites of SCNC biocomposite, three fundamental thermodynamic parameters were investigated viz. the Gibbs free energy of adsorption (ΔG^**0**^), the enthalpy change (ΔH^**0**^), and the entropy change (ΔS^**0**^)^74–76^. The thermodynamic parameters ΔG, ΔS and ΔH for this adsorption process were determined by using the following equations: Eqs. (12–14)

12 $$\Delta {\text{G}}^{{\mathbf{0}}} = - {\text{RT}}\;{\text{lnKd}}$$

13 $${\text{K}}_{{\text{d}}} = {\text{q}}_{{\text{e}}} /{\text{C}}_{{\text{e}}}$$

14 $$\ln K_{d} = \frac{{ - \Delta H^{o} }}{{RT}} + \frac{{\Delta S^{O} }}{R}$$

where C_e_ is the adsorbate equilibrium concentration in (mg L^− 1^), C_o_ is the adsorbate initial concentration in (mg L^− 1^ ), and T is the temperature in (K). R is the universal constant in (J K^− 1^mol^− 1^), while K_c_ stands for the dimensionless distribution coefficient.

By plotting log K_c_ versus 1/T, as shown in Fig. 13b, allowing for the determination of thermodynamic parameters like ΔH^**0**^ and ΔS^**0**^ and the findings are listed in Table 4.

The positive ΔH^**0**^ value (46.06 kJmol^− 1^) suggested an endothermic nature of the adsorption process. The positive value of ΔS^**0**^ (169.107kJmol^− 1^K^− 1^) indicated the increase in the randomness of the SCNC biocomposite/dye solutions interface during adsorption process. The negative values of ΔG^**0**^ (-4.718, -5.748, -7.320 and − 10.64 kJmol^− 1^ at 298, 308, 318 and 333 K), suggest that the adsorption process is feasible and spontaneous in nature. It was also observed that with an increase in temperature, the value of ΔG^**0**^ decreases, which indicated that the sorption processes was spontaneous and becomes more thermodynamically favorable with an increase in temperature.

**Table 4:** Thermodynamic parameters for the adsorption of CR on SCN &SCNC biocomposites.

Sample	K_c_				ΔG^0^ (kJ/mole)				ΔH^0^ (kJ/mole)	ΔS^0^ (J/mole K)
	298.15 K	308.15 K	318.15 K	333.15 K	298.15 K	308.15 K	318.15 K	333.15 K		
SCN	1.051	---	2.325	4.121	-0.214	---	-2.23	-3.92	32.16	108.24
SCNC	6.710	9.427	15.920	46.619	-4.718	-5.748	-7.320	-10.64	46.06	169.107

#### Influence of ionic strength

The influence of ionic strength on the removal CR from aqueous solution was studied at optimum condition as shown in Fig. S3. It can be seen that the removal % of CR increases from 97 to 99.5% with increasing the concentration of sodium chloride from 0.01 M to 0.16 M. Addition of sodium chloride salt causes that, the CR dye solution aggregation which leads to decrease in the solubility. Aggregation of CR dye molecules, result in promotion the adsorption of the CR dye solution, and hence increasing in the dye removal efficiency^76,77^. Also, when the ionic strength increases, cations from the salt (Na^+^) increases the positive charge of the adsorbent surface thus increases the electrostatic attraction between the negative dye and positive adsorbent surface resulting in an increase in adsorption of CR.

#### Effect of co-existing ions on CR adsorption onto SCNC biocomposite

The effect of various commonly existing ions such as bicarbonates, phosphates, carbonates, nitrates on the adsorption of CR onto SCNC is presented in Table 5. The adsorption efficiency(R,%) decreases with increasing the concentration of these ions, where carbonates exhibited the minimum effect.

**Table 5:** Effect of co-existing ions on the adsorption efficiency of 50 mgL^− 1^ CR using SCNC.

Co-existing ions	Concentration, mgL^− 1^	Removal, *R* %
Bicarbonate (HCO_3_^−^)	1	98.80 ± 1.516
	5	95.66 ± 1.394
	10	90.88 ± 1.744
	50	63.88 ± 0.029
Carbonate (CO_3_^2−^)	1	99.22 ± 0.739
	5	97.51 ± 1.234
	10	95.34 ± 1.456
	50	71.51 ± 1.717
Nitrate (NO_3_^−^)	1	97.18 ± 0.026
	5	95.46 ± 1.034
	10	93.18 ± 1.089
	50	68.16 ± 1.426
Phosphate (PO_4_^3−^)	1	98.44 ± 1.010
	5	95.34 ± 1.404
	10	92.27 ± 1.320
	50	61.27 ± 1.213
Sulfate (SO_4_^2−^)	1	93.22 ± 0.066
	5	90.59 ± 1. 506
	10	88.50 ± 0.090
	50	58.59 ± 1.096

#### Desorption and regeneration experiments

Reuse of spent adsorbent and recovery of adsorbate will make the treatment process economical and it is necessary to regain the adsorbent after adsorption. By studying the mentioned solution in the desorption efficiency we found that, absolute ethanol, nitric acid, hydrochloric acid and sodium hydroxide, sodium-bicarbonate concentrations between (0.01 to 0.1 M) have very weak effect on the desorption of the loaded SCNC-CR complex as shown in Table 6. The best solution for the desorption of SCNC-CR was a mixture of (0.01 N of sodium-bicarbonate and 0.05 N sodium hydroxide) where the desorption efficiency% reaches 91.63%. Furthermore, the reusability evaluation revealed that the sorbent can be used repeatedly for 5 times with a minimal decrease in the adsorption capacity, as shown in Table 7.

**Table 6:** Recovery percent for CR with the different eluting solutions.

Eluting solution	De%
(0.01) mol/L NaOH	3.9
(0.05) mol/L NaOH by heating	3.53
(0.1) mol/L NaOH by heating	-
(0.03) mol/L Na_2_CO_3_	14.78
(0.01) mol/L Na_2_CO_3_+ (0.01) mol/L NaOH by heating	2
(0.05) mol/L Na_2_CO_3_+ (0.01) mol/L NaOH by heating	14.45
(0.01) mol/L Na_2_CO_3_ (0.05) mol/L + NaOH by heating	91.63
(0. 1) mol/L Na_2_CO_3_ + (0. 1) mol/L NaOH	0.18

**Table 7:** Repeated adsorption of CR by SCNC sorbent (0.025 g), time of shaking 200 min.

Cycle number	1	2	3	4	5
Recovery (%)	91.63	91.33	90.54	89.77	89.0

#### Applications

Optimized experimental conditions were performed on real samples to evaluate the efficiency of SCNC for CR dye sorption. A calibration curve was generated using known standard solutions. Approximately (1.0 L) of standard solution of CR was processed under the optimal conditions described above. Analytical water samples were collected from laboratory tap water at Mansoura University, Nile water from Mansoura city, and seawater from Alexandria city. The analytical results are shown in Table 8. CR dye was not detected in all samples. Recovery was examined in samples spiked with known amounts of CR dye. The yields achieved were 98.00-99.42%. These results demonstrate that SCNC biocomposite can be applied to remove anionic dyes in real water samples.

**Table 8:** Analytical results of sorption of CR anionic dye (µgml^− 1^) in real water samples using SCNC biocomposite (*n* = 5).

Sample	Spiked (µgmL^− 1^)	Measured (µgmL^− 1^)	Recovered (µgmL^− 1^)	Recovery (%)	RSD,%*
Tap water	0.00	0.00	0.00	0.00	--
	50	0.5	49.5	99.0	1.40
	100	1.8	98.2	98.2	2.30
Nile water	0.00	0.00	0.00	0.00	--
	50	0.93	49.07	98.14	1.89
	100	2.00	98	98	1.45
Sea water	0.00	0.00	0.00	0.00	--
	50	0.33	49.67	99.34	2.50
	100	1.27	98.73	98.73	1.76

*RSD relative standard deviation.

#### Evaluation of the performance of SCNC biocomposite

Comparison studies of adsorption of CR dye by other materials have been given in Table 9. The present material has good adsorption capacity than others. So, the SCNC biocomposite can suitably be applied for wastewater treatment.

**Table 9:** Comparison of maximum sorption capacity of CR by proposed SCNC biocomposite with some of the previously published articles.

Adsorbent	q_e_, mg.g^− 1^	References
Kaolin (clay materials)	5.44	[78]
Acid activated red mud	7.08	[79]
Montmorillonite	12.70	[80]
Ball-milled sugarcane bagasse	39.8	[81]
Magnetic core–manganese oxide shell	42.0	[82]
Chitosan/montmorillonitenanocomposite	54.52	[80]
Ca-bentonite	107.41	[83]
SCNC biocomposite	170	This study

#### Plausible mechanism of CR adsorption onto SCNC biocomposite

**Fig. 14:**
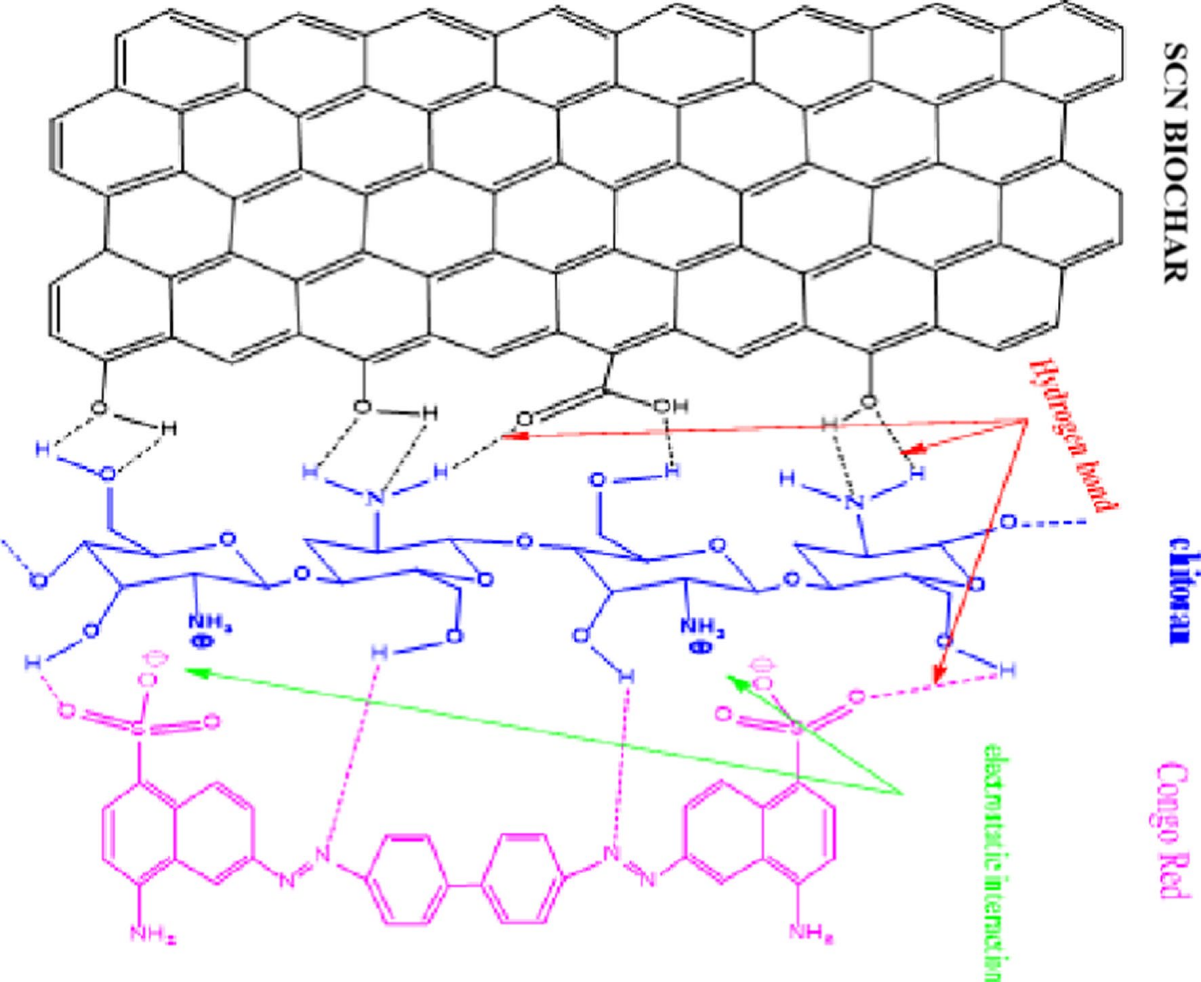
Plausible mechanism of CR adsorption onto SCNC biocomposite

The FTIR spectral analysis, as discussed earlier, explains that the SCNC surface contains a variety of functional groups, such as -COOH and -OH, that can function as active adsorption sites. However, following adsorption, these functional groups’ vibrational bands experienced a blue or red shift in response to the adsorption of CR. This demonstrated that the CR dye adsorption process involved the active functional groups. The several forms of interactions, such as pore filling, pi-pi interactions, electrostatic attractions, and H-bonding, can be attributed to the CR dye adsorbed onto the surface of the SCNC biocomposite. Since the length of CR is lower (18.3 Å) than the pore size (26.6 Å) of the SCNC, CR dye can physically enter these pores and be extracted from the solution.The second is electrostatic attraction; the CR dye molecule was negatively charged at pH < pH_PZC_, and the surface of the SCNC biocomposite was positively charged, resulting in electrostatic attraction. This could be due to the electrostatic interaction between protonated amino (NH^3+^) groups of the SCNC and the (-SO_3_^−^) sulfonated group found in CR dye.The third is pi-pi interactions; since both the CR dye and the SCNC biocomposite had hexagonal benzene rings, hydrophobic attraction developed as a result of the dye adhering to the SCNC surface. H-bonding is the final interaction that is feasible; the -OH groups of SCNC biocomposite formed an H-bond with the CR dye’s N heteroatom. Figure 14 summarizes these potential interactions between the surface of the SCNC biocomposite and the CR dye. Additionally, other researchers observed comparable observations^84–86^.

## Conclusion

**Fig. 15:**
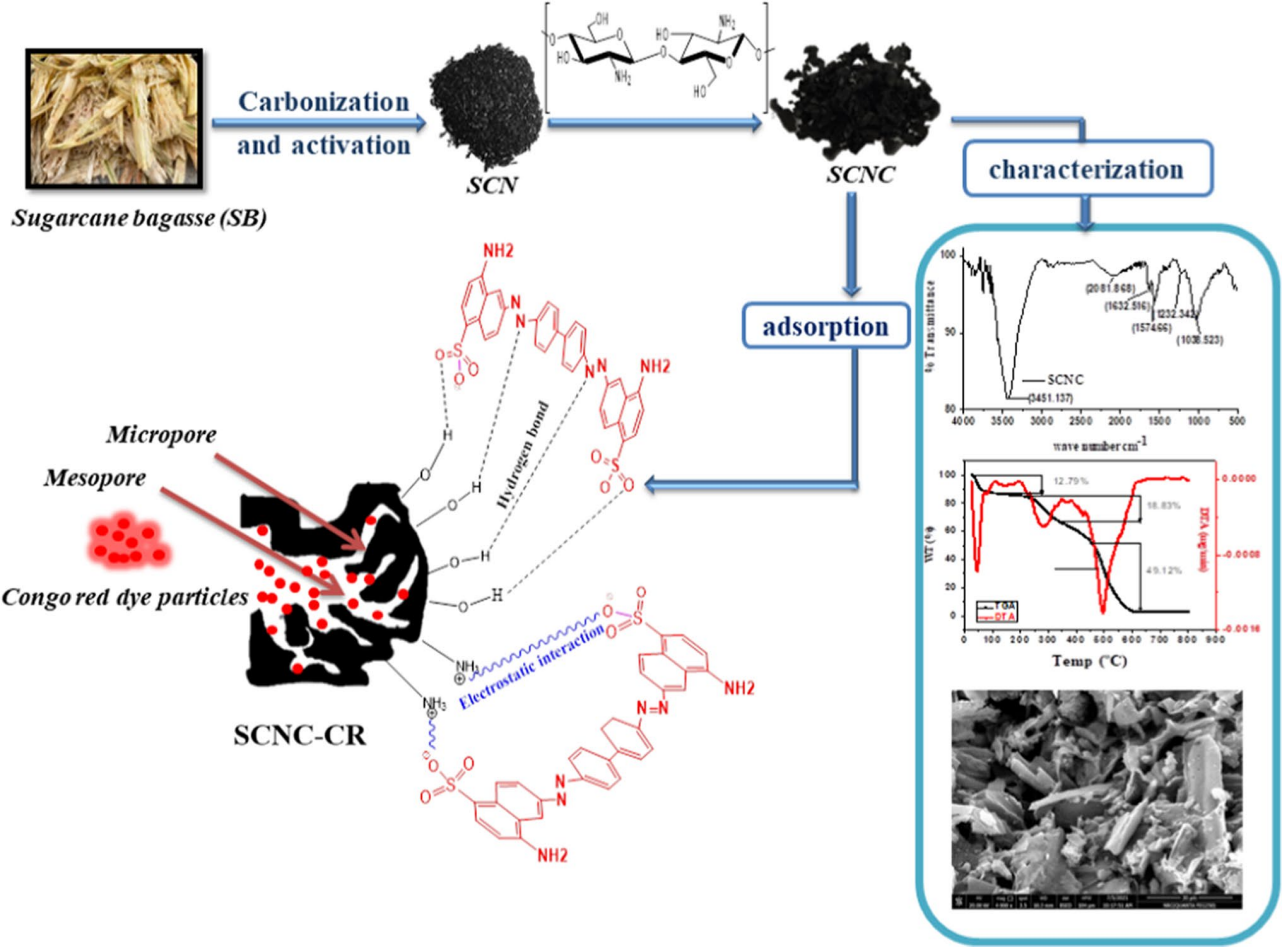
Synthesis, characterization and adsorption of CR onto SCNC biocomposite.

This study demonstrated the excellent adsorption capacity of the synthesized chitosan impregnated sugarcane bagasse biochar (SCNC) biocomposite for the removal of CR dye from aqueous solutions. The prepared SCNC biocomposite was characterized using SEM, FTIR, and TGA-DTA, and various operational parameters were also studied. The results obtained under the optimum conditions showed that the prepared SCNC biocomposite has considerable removal percentages of 98.37% for CR dye. Because of the higher correlation coefficient (R^2^ ≥ 0.999) and lower error functions, the equilibrium CR adsorption isotherms for a single-dye system fit Langmuir and the PSO model.The maximum adsorption capacity(q_max_) for CR was found to be 170 mgg^− 1^; a value that’s comparable or better than various adsorbents previously reported for adsorption of CR. The adsorption of CR was influenced by initial concentration of dye, contact time, dosage, pH and temperature. Thermodynamic studies showed that the adsorption was spontaneous and endothermic with an increase in the randomness of the SCNC biocomposite/dyes solutions interface. The desorption results showed that above 90% of CR were desorbed by 5mL of 0.01 N of NaHCO_3_ and 0.05 N NaOH in the first cycle. The regeneration studies showed that the SCNC biocomposite can be regenerated up to 5 cycles successfully. Therefore, the newly synthesized SCNC biocomposite can be utilized as a promising green adsorbent for the removal of CR anionic dyes from aqueous solution very economically. The synthesis, characterization of the SCNC biocomposite is schematically represented in Fig. 15.

## Electronic supplementary material

Below is the link to the electronic supplementary material.

Supplementary Material 1

## Data Availability

The authors declare that the data supporting the findings of this study are available within the paper and its Supplementary Information files.
