# Supplementary material for: Chitosan impregnated sugarcane bagasse biochar for removal of anionic dyes from wastewater
Source: Sci Rep. 2024 Nov 7;14:27097. doi: 10.1038/s41598-024-77708-9 (PMC11543815; doi:10.1038/s41598-024-77708-9)
Supplement: Supplementary file 1 — Supplementary Material 1 [file 41598_2024_77708_MOESM1_ESM.docx]

**Supporting data**

**Chitosan impregnated sugarcane bagasse biochar for removal of anionic dyes from wastewater**

**Magda Akl**^1*^**and Asmaa Serage^1^**

^1^ Department of Chemistry, Faculty of Science, Mansoura University, Mansoura 35516, Egypt

*Corresponding author

Prof Dr. Magda Ali Akl

Professor of Analytical Chemistry,

Department of Chemistry,

Faculty of Science, Mansoura University,

Mansoura 35516, Egypt

magdaaakl@yahoo.com

| 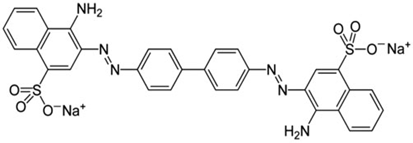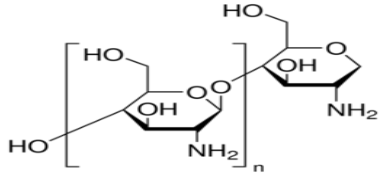   1. CR (b) Chitosan |
| --- |
| **Figure S1: chemical structure of (a) CR & (b) chitosan** |

| 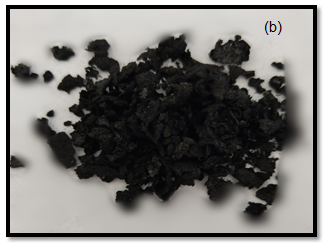 | 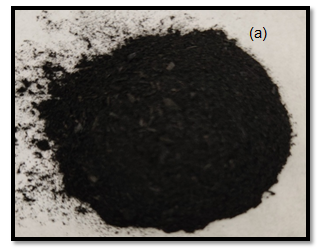 |
| --- | --- |

**Figure S2 Optical images of (a)SCN and (b) SCNC**

|  |
| --- |

**Figure S3: Effect of ionic strength on SCN and SCNC samples**
